# Faculty Retreats in Academic Medicine: Tutorial

**DOI:** 10.2196/71622

**Published:** 2025-11-03

**Authors:** Rachel Skains, Julie Brown, Erin F Shufflebarger, Justine McGiboney, Sherell Hicks, Laine McDonald, Katherine B Griesmer, Christine Shaw, Emily Grass, Marie-Carmelle Elie, Lauren A Walter

**Affiliations:** 1 Department of Emergency Medicine, Heersink School of Medicine, University of Alabama at Birmingham, 519 19th St S, Birmingham, AL, 35223, United States, 1 205-975-8837

**Keywords:** faculty development, faculty retreat, academic medicine, organizational leadership, medical education

## Abstract

Faculty development is a cornerstone of academic medicine, supporting personal growth, professional advancement, and departmental effectiveness across all stages of a faculty member’s career. Among the tools available, faculty retreats have increasingly emerged as a high-impact strategy to foster collaboration, advance strategic planning, and address individual and collective goals in a structured, reflective setting. While retreats are widely used in other sectors, practical guidance tailored to the academic medicine context remains limited. This tutorial offers a comprehensive, step-by-step framework for planning and implementing faculty retreats within academic departments. Key elements of effective retreat design are outlined, including (1) conducting a preretreat needs assessment to align goals with faculty priorities, (2) selecting an appropriate format (eg, in-person or hybrid), (3) fostering psychological safety to enhance participation, and (4) using facilitation techniques that promote inclusive dialogue and actionable outcomes. The tutorial also emphasizes logistical considerations, such as agenda design, timing, and participant engagement strategies, alongside mechanisms to ensure follow-up and accountability after the retreat. In addition to highlighting common barriers, such as resource limitations, scheduling constraints, and engagement disparities, the tutorial provides practical solutions drawn from real-world examples in academic medicine. By integrating thoughtful planning, evidence-informed facilitation, and postretreat follow-through, faculty retreats can serve as transformative experiences that support both individual development and departmental cohesion. This resource aims to fill a gap in the literature by equipping leaders in academic medicine with a structured approach to designing, executing, and sustaining the benefits of faculty retreats.

## Introduction and Background

As faculty development in academic medicine has evolved, it now emphasizes career-long growth and attention to both individual and departmental dynamics [1,2]. Faculty retreats have emerged as a valuable tool to enhance performance and institutional effectiveness by bringing faculty together outside routine settings for collaboration, strategic planning, and professional development. Retreats also offer a structured space to address challenges, build consensus, resolve conflicts, and foster collegiality (Textbox 1; [3-12]).

Textbox 1. **Benefits of faculty development retreats.**

**Skill enhancement**

Retreats provide dedicated time for faculty to engage in specific professional development activities [4,5].

**Improved collaboration and networking**

Retreats foster interdisciplinary collaboration and networking among faculty, leading to new research partnerships, coteaching opportunities, and the sharing of best practices [5,6].

**Reflection and constructive feedback**

Faculty retreats can offer time for reflection on teaching, research, and administrative responsibilities. Structured feedback from peers during retreats helps faculty identify areas for improvement and develop actionable plans to enhance their performance [7].

**Burnout prevention and well-being**

Retreats provide a break from the daily pressures of academic and clinical life, allowing faculty to focus on self-care and stress management. This can lead to improved well-being, reduced burnout, and increased job satisfaction, which positively affect performance [8].

**Strengthened culture and community**

There is a strong correlation between team building and company or department culture. The mutual collaboration that happens over the course of team-building exercises fosters a sense of community. This, in turn, helps department culture to develop and evolve smoothly and in a way that is true to the departmental vision [9].

**Innovation and development**

Faculty retreats often serve as spaces for brainstorming and developing innovative teaching strategies, curricular changes, new research, or quality improvement ideas [10].

While retreats have long been common in the business world, their broader use in academic medicine is more recent [13]. However, practical guidance for planning and implementing faculty retreats remains limited and fragmented. This tutorial offers a comprehensive overview of the purpose, value, and implementation of faculty retreats within academic departments, highlighting current trends and best practices.

## Solution

### Faculty Retreat Implementation Strategies

#### Preretreat Preparation: Needs Assessment

Conducting a needs assessment prior to the faculty retreat ensures that the most pressing issues are addressed, the goals of the intended group are aligned, and the participants are engaged effectively [14]. It is important to communicate the objectives of the needs assessment to the faculty prior to response solicitation by clarifying the purpose. It should be explained clearly that the data are intended to be used to direct faculty development objectives, including retreat planning. The needs assessment should gather information to achieve these goals. Further, the scope of the needs assessment should be carefully considered, whether it will focus on individual faculty members, a whole department, or the institution as a whole. This helps target the assessment appropriately.

It is important to ensure all faculty members who will be the intended participants of the retreat have an opportunity to contribute to the needs assessment. This increases buy-in and ensures the retreat meets their expectations. If the retreat involves interdisciplinary work or collaborations with external partners, gather input from relevant stakeholders to understand their expectations and needs as well. Finally, the needs assessment participants may constitute a larger group than those subsequently engaged in a future retreat, as the data may be used more broadly for additional faculty development efforts.

Consider the use of multiple methods for needs assessment data collection [15]. A combination of qualitative and quantitative methods provides a more robust picture of faculty needs by gathering diverse perspectives. This can include surveys and questionnaires (eg, Google Forms, SurveyMonkey (SurveyMonkey Inc), Qualtrics (Qualtrics International Inc), etc, for easy distribution and analysis). Use a mix of open-ended and multiple-choice questions to cover various areas (Textbox 2). Finally, ensure that survey responses are anonymous or use anonymous identifiers to provide distinction between responders, if needed.

Textbox 2. **Example of preretreat needs assessment questions.**

- What are the main challenges you face in your teaching or research?
- What are your goals for the upcoming academic year?
- What topics would you like to see covered at the retreat (eg, leadership development, team building, teaching strategies, and research collaboration)?
- How do you feel about the current team dynamics within the department/faculty?
- How can the retreat best support your professional development?
- Ranked Priorities: Ask faculty to rank the importance of potential retreat topics, such as:

- Well-being and self-care/self-compassion
- Mentorship and sponsorship
- Negotiating effectively
- Effective communication
- Strategic planning
- Team building
- Increasing scholarly activity (eg, research productivity)
- Leadership and management
- Promotion and tenure

Needs assessment data collection may also involve interviews or focus groups [16]. One-on-one in-depth interviews with leadership, faculty leaders, and administration can obtain deeper insights into institutional or departmental strategic priorities. This ensures that the retreat is aligned with larger institutional and departmental goals. Ask about specific challenges within departments, faculty development gaps, and potential solutions. Use these interviews to uncover nuanced issues that might not emerge in a survey. Organize small focus groups to encourage open discussion among faculty members. This is particularly useful for gathering collaborative input on broader issues, like curriculum design or faculty culture. Consider having a neutral facilitator to ensure open, honest dialogue.

In addition to solicited participant input, also consider reviewing objective institutional or departmental data. This can include performance data, such as performance metrics, teaching evaluations, faculty publication records, and other key indicators. This data can help identify key areas where faculty need support, such as improving student engagement or increasing research output. Review any existing data from faculty climate surveys or teaching evaluations to identify patterns or concerns that need to be addressed during the retreat. Finally, if available, analyze feedback from previous retreats or faculty development initiatives to identify ongoing or unresolved issues.

Once the needs assessment is obtained, identify key themes and prioritize needs [17]. Organize the feedback into categories, such as personal development needs, leadership skills, work-life balance, mentoring, etc. Before the retreat, share a summary of the needs assessment findings with faculty. This transparency builds trust and helps participants understand how their input has shaped the retreat agenda. Once developed, provide a draft of the retreat agenda, highlighting how it addresses the needs and priorities gathered from the assessment.

Finally, consider the needs assessment timeline. It should be conducted well enough in advance of the anticipated retreat to provide ample time for analysis and planning but not so far ahead of time that the data obtained and reviewed are no longer timely or pertinent. By conducting a thorough needs assessment, the faculty retreat can be tailored to the specific needs of the group, which will increase engagement, relevance, and the overall success of the retreat.

#### Preretreat Preparation: Setting Retreat Goals and Objectives

A comprehensive consideration of needs assessment data (see above) should identify and prioritize specific objectives. By addressing common areas of interest and import, clear and meaningful goals and objectives can be set to guide the retreat’s agenda and outcomes [18]. In addition, early engagement of key participants, including faculty leaders and key stakeholders, will ensure that the retreat’s objectives align with the broader mission and vision of the institution or department. Consider that retreat funders may impart influence on a retreat’s scope and attempt to align an instructed scope with identified needs assessment priorities to improve the effectiveness of the retreat.

Finally, create SMART (Specific, Measurable, Achievable, Relevant, and Time-bound) goals to provide a clear roadmap for retreat planning and postretreat follow-up [19]. These goals help ensure that retreat objectives are not only aspirational but also operational. For example, instead of a vague aim, such as “foster collaboration,” a SMART goal would be: *“*Improve cross-departmental collaboration by initiating at least three joint research proposals within the next academic year.*”* Using SMART goals helps organizers and participants align around shared expectations and provides a framework for evaluating success. Specific goals target a defined area for improvement; Measurable goals allow for tracking progress; Achievable goals ensure feasibility given available resources; relevant goals align with broader institutional priorities; and Time-bound goals set a clear deadline. During planning, facilitators should work with stakeholders to codevelop 2-3 SMART goals that reflect the most pressing needs and strategic aims of the department or institution. These goals can then guide session design, inform postretreat evaluation metrics, and promote accountability by linking retreat outcomes to tangible follow-up actions.

Prioritization of objectives will be required if the needs assessment identified several themes or areas of interest. Differentiate between objectives that can be addressed during the retreat versus those that require ongoing effort. Retreat goals can include long-term objectives, but given time constraints, the retreat may only be a start or a contributing component to that objective. Aligning the retreat content with long-term objectives can be effective from a strategic planning perspective. Finally, focus on a manageable number of objectives to avoid overwhelming participants; consider what is feasible in the retreat time allotted.

#### Preretreat Preparation: Resource Allocation

Determining resource allocation for a faculty retreat involves careful planning to ensure that funds, time, and human resources are used efficiently to meet the retreat’s objectives [20]. One of the most important first steps is to set the budget, considering which elements are critical (resources essential to the success of the retreat) versus optional. Review the available budget from the department, institution, or external grants. Be sure to account for all funding sources and restrictions (Textbox 3).

Textbox 3. **Potential retreat costs for budget consideration.**

**Venue costs**

Space rental for meetings, workshops, and team activities

**Meals and refreshments**

Catering for meals, snacks, and coffee breaks

**Accommodation (if overnight)**

Lodging for multiday retreats

**Travel expenses**

Transportation costs (eg, shuttles, mileage reimbursement, train, or flight tickets)

**Facilitator or speaker fees**

Costs for external facilitators, speakers, or consultants

**Workshop materials**

Supplies for activities (eg, printed materials, flipcharts, and projectors)

**Recreational activities**

Funding for team-building exercises, social activities, or outings

**Miscellaneous**

Any additional costs (eg, technology support, insurance, and contingency funds for unexpected expenses)

**Plan for contingencies**

Buffer for unexpected costs. Set aside a portion (typically 5%-10%) of the overall budget as a contingency fund for unforeseen expenses, such as last-minute speaker fees, additional supplies, or travel delays

In addition to financial costs, consider human resources allocation, including the creation of a retreat organizing committee. Identify staff or faculty members who will be responsible for planning, logistics, and facilitating the retreat. Ensure these roles are clearly defined, including responsibilities for agenda setting, coordinating logistics with the venue and vendors, and managing participant communication. If the retreat requires significant logistical support, allocate part of the budget to administrative staff, event planners, or student assistants to assist with setup, note-taking, or technical support during the event.

#### Preretreat Preparation: Selecting Participants and Faculty

Selecting participants for a faculty retreat involves careful consideration to ensure that the right mix of individuals is involved, based on the retreat’s purpose and goals (see above) [21]. Based on retreat objectives, consider prioritizing a balanced representation. This can involve fostering diversity in experience by engaging faculty across the career spectrum, from early-career to senior members, to enrich perspectives and strike a balance between tradition and innovation. This can also include cultural diversity to ensure background and social diversity to promote inclusive discussions and a wide range of viewpoints. Finally, tailor role-specific involvement to the retreat’s focus by including teaching and education faculty for curriculum development, research-active faculty and grant managers for research goals, and both faculty and professional staff for career growth discussions.

When appropriate, encourage voluntary participation. Identify motivated participants by soliciting a call for interest. Send an open call to faculty and allow those with an interest in the retreat’s objectives to self-nominate. However, a more selective invitation process may be required in some cases to ensure that the right people are present, especially for leadership-focused retreats.

Keep the group size manageable, typically between 15 and 25 participants, depending on the format. On some occasions, an even smaller cohort may be preferred, no more than a dozen, to ensure all voices are heard and universal participation is feasible. Smaller groups are more conducive to intimate, productive discussions, while larger groups can handle broader, more diverse topics. For larger retreats, plan for breakout sessions that enable smaller, focused discussions among participants. If relevant, consider inviting external facilitators or industry experts who can offer outside perspectives and guide discussions more effectively. Finally, account for logistical and financial constraints incurred by the number of retreat participants, including travel, budget constraints, and scheduling availability.

#### Preretreat Preparation: Choosing Location and Setting

Choosing a location for a faculty retreat involves several key considerations to ensure that the setting supports the retreat’s goals and fosters collaboration, relaxation, and productivity [22]. For example, if the focus is academic, such as strategic planning, curriculum development, or research collaboration, a location with quiet meeting spaces and minimal distractions might be best. If the focus is team building or bonding, consider locations that offer outdoor activities or team-building opportunities, such as nature resorts or retreat centers. Proximity and accessibility should be considered. Consider a central location within a reasonable distance easily accessible by car, train, or public transport, especially if it is a 1- or 2-day event or if participants are coming from multiple campuses. If it is remote, ensure there are clear directions and transport options with ample parking for faculty members that are driving.

Regarding the actual meeting space and facilities, again consider the size of the group. The venue should have meeting rooms that accommodate the entire group comfortably. Look for spaces with flexible seating arrangements, good acoustics, and appropriate lighting. Also, consider technology needs and ensure that the facility can support presentations or workshops with the necessary audiovisual equipment (eg, projectors, screens, microphones, and Wi-Fi). If smaller group discussions or breakout sessions are planned, look for venues with multiple meeting rooms or areas conducive to group work. If it is a multiday retreat requiring an overnight stay, ensure the location offers comfortable amenities and accommodations for faculty. Confirm dining options available at the facility, including considerations for dietary restrictions. If incorporating relaxation or leisure time, look for venues that offer recreational amenities, such as hiking trails, swimming pools, or wellness facilities. Finally, consider the cost; a venue must fit the budget without compromising on essential needs. Consider that some universities or institutions have partnerships with certain venues or offer discounts for academic groups.

When choosing a location, group dynamics, inclusivity, and environment should also be considered. Consider accessibility for faculty members with mobility issues, and ensure that spaces accommodate various needs (eg, lactation room). If you have a diverse group, make sure the venue respects cultural differences, including dietary needs, religious observances, and inclusive spaces. Make sure the location is available for your preferred dates by planning early as popular retreat centers and venues often book up months in advance. Finally, ambience and environment are important elements to also consider for enhancing faculty engagement and collaboration. Consider seasonal considerations, as outdoor activities might be less enjoyable during rainy or cold seasons, and ensure the venue is prepared for inclement weather. Many faculty retreats benefit from being in scenic or natural settings (eg, mountains, lakesides, or parks) as they promote relaxation and creativity. Some institutions may prefer locations with cultural or academic significance, such as university-affiliated retreat centers, historical sites, or museums.

#### Preretreat Preparation: Structuring the Agenda and Activities

**Figure 1.**
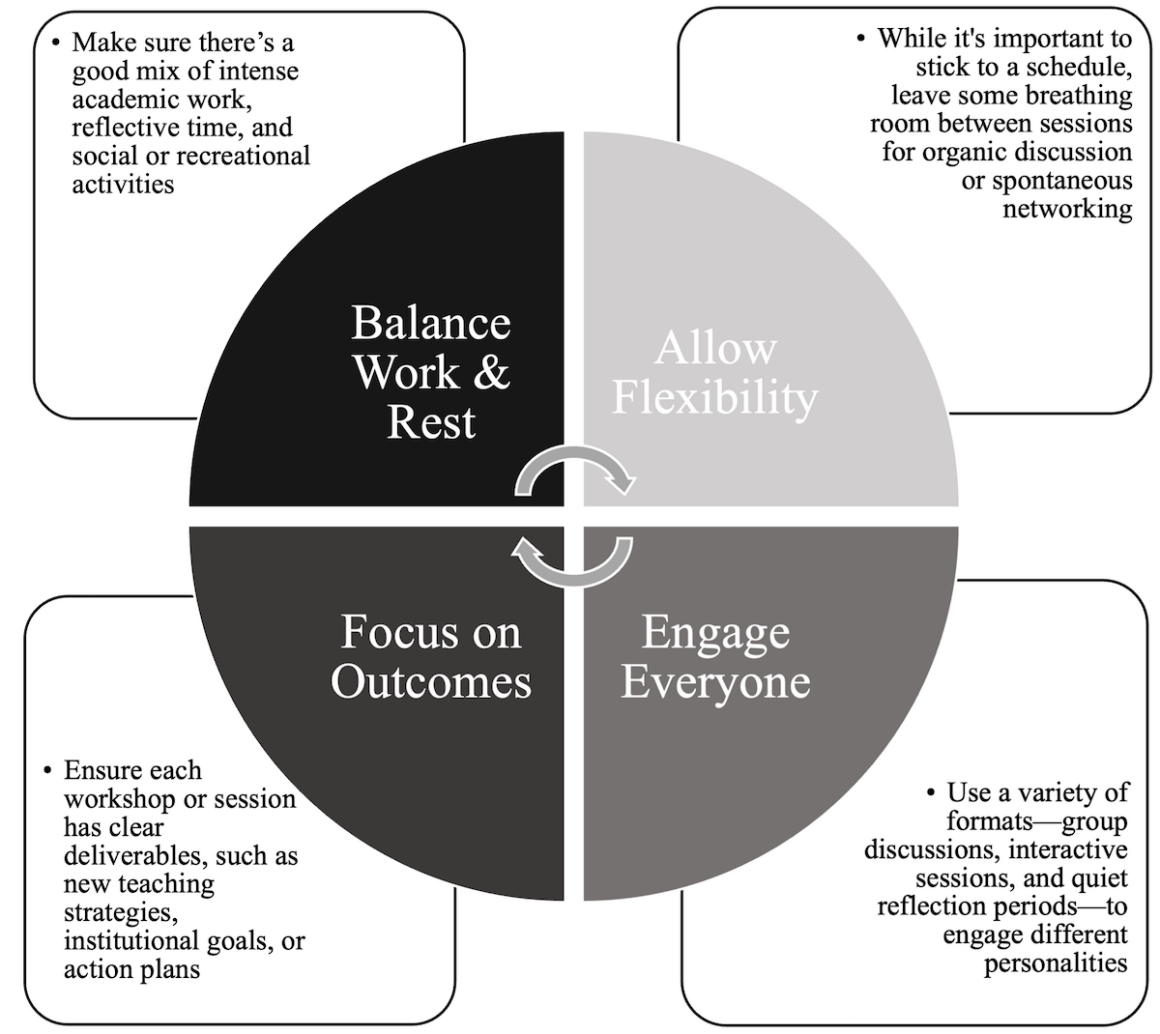
Key agenda design tips.

Before creating the agenda, establish the key objectives (see above). The agenda should be designed explicitly to meet these goals and reflected in any workshops, seminars, or team-building exercises (Figure 1; [23,24]). Allowing the identified key objectives to guide and define retreat content creates a clear path for a more meaningful faculty experience. While noncomprehensive, a list of potential retreat content has been provided in Textbox 4 along with an example retreat agenda (one and a half-day; Table 1).

Textbox 4. **Agenda content ideas.**

**Professional development workshops**

- Effective mentorship across career stages: Techniques for becoming or finding a mentor; case-based discussions.
- Navigating promotion and tenure: Department-specific guidance on academic advancement pathways.
- Time management for academic clinicians: Focused strategies on balancing clinical, teaching, and research responsibilities.
- Grant writing and funding strategies: Tips for National Institutes of Health/Health Resources and Services Administration (NIH/HRSA) submissions, developing specific aims, or finding pilot funds.
- Leadership skills for faculty: Training on conflict resolution, delegation, and leading teams.
- Work-life integration and wellness in academic medicine: Interactive session focused on reducing burnout and enhancing resilience.

**Culture-focused sessions**

- Implicit bias in clinical and academic settings: Facilitated discussion with activities for self-reflection and behavioral change.
- Building an inclusive department culture: Group exercises around shared values, microaggressions, and allyship.
- Health equity in research and education: Incorporating equity principles in curriculum, research, or patient care.

**Strategic planning and innovation**

- SWOT (strengths, weaknesses, opportunities, and threats) analysis breakout groups: Analyze departmental strengths, weaknesses, opportunities, and threats.
- Visioning and goal-setting workshops: Define shared goals for the next 1‐3 years using techniques like appreciative inquiry.
- Innovation challenge or hackathon: Small groups generate and pitch new ideas (eg, curriculum and QI; quality improvement projects).
- Artificial intelligence (AI) in academic medicine (opportunities and risks): A future-facing panel or demo of tools for teaching, research, or clinical decision-making.

**Team-building activities**

- “Mission Possible” challenge: Groups solve department-relevant scenarios collaboratively (eg, resource allocation or onboarding plans).
- Human bingo or speed networking: Fast-paced way to connect across faculty and discover shared interests.
- Story circles or narrative medicine exercise: Faculty share brief, structured stories on meaningful clinical or academic experiences.
- Escape room or puzzle challenge (in person or virtual): Promotes collaboration and informal interaction.
- Personality type-based communication exercise (eg, MBTI; Myers-Briggs Type Indicator, StrengthsFinder, and DISC; Dominance, Influence, Steadiness, and Conscientiousness) to explore working styles and improve collaboration.

**Webinars or prerecorded content**

- “Academic Medicine in 2030 and Beyond” (invited guest speaker): Shown at the retreat or asynchronously, followed by discussion groups.
- NIH/funding agency updates: Presented by the internal grants office or invited program officers.
- Faculty wellness microlearning modules: Paired with breakout reflections or personal wellness planning.

**Reflection and closure activities**

- Commitment cards: Participants write one commitment they will take forward, which is collected and mailed to them months later.
- Gallery walk: Groups post ideas/goals on flip charts around the room and rotate to add or comment.
- Shared success board: Space to acknowledge team wins and individual accomplishments over the past year.

**Table 1.** Example retreat agenda (one and a half days)*.*

Day, theme, and time	Session	Description
Day 1: faculty retreat (full day); theme: Advancing Together: Strategy, Scholarship, and Community		
8-8:30 AM	Arrival and Breakfast	Light breakfast and coffee Informal networking
8:30-9 AM	Welcome and Overview	Opening remarks by the Chair or Vice Chair Goals for the retreat
9-10:15 AM	Strategic Visioning Workshop	Facilitated SWOT^a^ analysis and goal-setting exercise in breakout groups
10:15-10:30 AM	Break	—^b^
10:30 AM-12 PM	Faculty Development Breakouts	Choice of concurrent sessions Grant writing tips and resources Promotion pathways and CV^c^ building Burnout prevention and work-life integration
12-1:00 PM	Lunch (working or social)	Optional discussion tables (eg, “Mentorship in Medicine” and “DEI^d^ in Action”)
1-2:30 PM	Team Challenge: Mission Possible	Small-group scenario activity focused on real departmental challenges (eg, onboarding, DEI, and teaching loads)
2:30-2:45 PM	Break	—
2:45-4 PM	Psychological Safety and Inclusive Culture Workshop	Facilitated session with case examples and strategies for fostering trust and belonging
4-4:30 PM	Gallery Walk: Big Ideas Board	Faculty rotate among flipcharts capturing ideas from earlier sessions and add input
4:30-5 PM	Day 1 Wrap-Up and Preview	Recap of themes, reflection exercise, and preview of day 2
5-6:30 PM	Optional Social Hour	Informal reception or outdoor gathering (may include dinner or group activity)
Day 2: Faculty retreat (half day); theme: From Ideas to Action		
8-8:30 AM	Breakfast and Networking	Casual start with time for reflection
8:30-9:45 AM	Lightning Rounds: Faculty Innovations	5-minute presentations by faculty on projects, teaching methods, or QI^e^ work
9:45-10:30 AM	Small Group Action Planning	Based on day 1 priorities (eg, mentorship, wellness, and diversity), groups draft next steps
10:30-10:45 AM	Break	—
10:45-11:30 AM	Department Town Hall	Leadership addresses submitted questions, strategic updates, and resource planning
11:30 AM-12 PM	Closing Reflections and Commitment Cards	Faculty write one goal or takeaway Cards mailed to them in 3-6 months
12 PM	Adjourn	Optional lunch or grab-and-go meal

a SWOT: Strengths, Weaknesses, Opportunities, Threats.

b Not applicable.

c CV: curriculum vitae.

d DEI: diversity, equity, and inclusion.

e QI: quality improvement.

##### Conducting the Retreat

When it comes to conducting the retreat, several elements can ensure that things run smoothly. Primarily, identify a retreat coordinator, typically someone on the planning committee involved in the retreat planning process and familiar with all the retreat elements and plans. These individuals, a faculty or staff member, will be responsible for monitoring the retreat agenda, timekeeping, and serving as a day-of contact for any outside speakers, moderators, or vendors. In addition, these individuals should have the capacity or resources to troubleshoot any potential hiccups, technology-related or otherwise. Depending on the size and scope of the retreat, delegation of specific roles to additional individuals may be required. Clear responsibilities, set ahead of time, allow for smooth session delivery and a more effective retreat.

Another element to consider while conducting the retreat is ongoing assessment of active engagement. Even with careful preretreat needs assessment analysis and planning, engagement can wax and wane during any retreat. As mentioned previously, incorporating a mixture of interactive sessions, small-group discussions, and hands-on workshops to maintain energy and involvement can make engagement drop-off less likely. Depending on retreat length, ample break time and access to refreshments can also assist with energy levels.

Finally, for sessions that might be more interpersonal, it can be important to recognize and acknowledge participant vulnerability and to normalize all responses and emotions. Creating a psychologically safe environment—where individuals feel comfortable expressing themselves without fear of embarrassment, rejection, or retribution—is foundational to fostering trust and vulnerability in retreat settings [25]. Psychological safety has been shown to improve team learning, engagement, and innovation in both health care and academic environments [26,27]. Faculty are more likely to share honest perspectives, disclose challenges, and collaboratively problem-solve when they perceive the environment as respectful, nonjudgmental, and supportive [28-33].

To cultivate psychological safety, facilitators should cocreate ground rules with participants, such as “assume good intent,” “confidentiality is expected,” and “all voices matter.” Setting norms early helps frame the retreat as a shared, inclusive space [34]. Facilitators can further normalize openness by modeling vulnerability themselves, sharing personal experiences or acknowledging areas of uncertainty, which signals that it is safe to take interpersonal risks.

Evidence-based facilitation techniques also enhance psychological safety. These include structured turn-taking (eg, round-robin formats), the use of anonymous input tools (eg, polling apps or sticky-note activities), and small group breakouts that lower the stakes for participation [35]. Active facilitation, such as naming group dynamics, gently redirecting dominant voices, and explicitly inviting quieter participants to share, further supports inclusion. Attending to emotional cues, validating contributions, and pacing sessions to allow reflection all contribute to a respectful tone and a sense of collective care.

By embedding these practices into the retreat design, organizers can create the conditions for open dialogue, team cohesion, and shared commitment to change. Further, anticipating common retreat challenges and potential solutions in advance can prove invaluable for both planners and participants (Textbox 5).

Textbox 5. **Common retreat challenges and solutions.**

**Budget constraints**

- Consider solicitation of either institutional or departmental support [28]. This funding source typically requires early engagement of high-level leadership and stakeholders along with well-defined anticipated outcomes. In the absence of a leadership-led retreat, this strategy may take initiative on the part of other faculty members.
- Self-funding or donation funding can be considered. Creation of a “Faculty Well-Being Fund” or “Development Fund” with voluntary donations can be a strategy option. While potentially effective, this approach could result in participation bias, with self-selected faculty or donors more likely to engage [29].
- Focus on local resources and simple activities, which do not require extensive budgets. Similarly, consider if a virtual retreat might be a sufficient option.
- Finally, consider holding the retreat at work during the workweek. While this limits the impact of an external environment, it might allow any existing budget to be nonetheless maximized [30].

**Schedule conflicts**

- Tagged, protected time can be considered. This is an often-used strategy for trainees, typically resident, retreats. This might be more challenging for academicians with 24-hour call or service schedules.
- Careful, extreme advanced planning and faculty notification, to avoid busy times of the year, whether seasonal or department-specific busier times, may help with turnout and engagement [33].
- Limiting the length of a retreat, generally recommended not to extend beyond 2 days, can also limit schedule conflicts [30].

**Participant engagement**

- When budget and logistics allow, consider separating the retreat site from work, as this may prevent work-related distractions and allow the group to stay on point [32].
- If the retreat is held at a destination or vacation location and families attend as well, consider limiting attendance at the actual retreat to faculty only, again to discourage distractions and promote focus. Family engagement may be appropriate at associated retreat social events at the organizers’ discretion.
- If occurring outside of usual work hours or expectations, consider whether provision of additional compensation may be possible. A notable limitation to consider here includes budget and introduction of participant bias.
- If the agenda allows, incorporation of social events is heavily encouraged. Scheduled social events can make it feel like an actual “retreat” from work. These events will also foster faculty camaraderie [30].
- Make sure there’s interest. Consider a preretreat needs assessment to gauge. Careful assessment of responses can avoid the subsequent expenditures of unnecessary efforts.

**Hybrid model considerations**

- To avoid participant inequity, adopt a “remote-first” mindset when planning content and facilitation strategies. Ensure all materials are digital and accessible in real time. Design activities that are inclusive for all participants, not just those in the room.
- Designate a cofacilitator or team member to advocate for and monitor remote participant inclusion.
- Intentionally structure breakout groups to mix in-person and virtual participants or keep them separate but equally resourced.
- Create informal spaces and scheduled time for social interaction across modalities.
- Include shared virtual coffee breaks, games, or team-building sessions where all participants engage in a similar experience, ideally via a single platform.
- Document key discussions and decisions in real time on shared platforms.

**Agenda Pitfalls**

- Avoid overpacking an agenda. If time is limited, concentrate on a singular objective directed by the needs assessment prioritization [33].
- Steer clear of a dull agenda. Avoid didactic-like structure [30].
- Postretreat neglect.
- Designate a “retreat secretary” to keep and distribute minutes. Establish an action item list, with deadlines, for identified postretreat interventions [33].
- Conduct a postretreat assessment; solicit feedback.

##### Postretreat Considerations

The conclusion of the retreat marks not the end, but the continuation of the organizers’ and leaders’ responsibilities. To be most effective, a good retreat involves solicitation and consideration of participant feedback. During the planning process, retreat objectives and outcomes should have been defined to outline how success will be measured. Tailor subsequent postretreat feedback instruments so that reviews can be considered in the context of the stated retreat goals and objectives. For instance, if team building is a goal, consider postretreat surveys to gauge improved collaboration. Feedback instruments can be survey-based and/or involve focus groups, like the process used for preretreat needs assessment data gathering. Consider both quantitative and qualitative feedback content. In addition, organizers should capitalize on the groundwork laid or the momentum gained by the retreat and seek future opportunities to weave positive retreat outcomes into continuous improvement and ongoing development (Multimedia Appendix 1).

To ensure that momentum continues beyond the retreat, organizers should establish structured follow-up mechanisms that translate insights into action. Begin by forming small working groups around priority areas identified during the retreat. These groups should be assigned clear deliverables and timelines and report progress during faculty meetings or through a shared digital platform. Designating retreat “champions” or coleads within each group can help maintain accountability and enthusiasm. Within 1 month post retreat, schedule a follow-up meeting to review early progress, reinforce shared goals, and recalibrate as needed. In addition, consider incorporating retreat themes into existing faculty development programs or launching targeted initiatives—such as peer mentoring circles, writing groups, or leadership development cohorts—aligned with retreat goals. Finally, build in evaluation checkpoints at 3, 6, and 12 months to assess progress, solicit feedback, and celebrate milestones. These actions foster sustained engagement and embed retreat outcomes into the academic fabric of the department.

##### Timeline Considerations

Although mentioned above, it deserves reiterating, as a final note in the planning process, that implementation of any retreat takes significant time, from organizing and conducting preretreat needs assessment through to evaluating and considering postretreat feedback and impact. When possible, at the onset, retreat planners should attempt to develop a “retreat timeline” (Textbox 6) that considers each aspect of the retreat development and implementation process. This timeline, which can doubly serve as a retreat checklist, should be retreat-specific and will be highly dependent on the anticipated breadth and expanse of the retreat. Dynamic timeline adjustments may need to be made pending results of preretreat assessments or other variables, including desired venue availability, participant work schedule, etc.

Textbox 6. **Example retreat timeline.**

**6 months (or more) in advance**

- Announcement of Faculty Retreat Intentions
- Open Invitation to Self-Nominate Organizing Committee

**4-6 months before**

- Preretreat needs assessment and analysis

**3-4 months before**

- Agenda creation
- Save-the-date creation to participants
- Speaker/moderator invitation
- Location and accommodation reservations

**1-2 months before**

- Agenda confirmation
- Formal faculty invitation and RSVP (répondez s'il vous plaît [French])

**1 week after**

- Immediate postretreat feedback solicitation and consideration

**1-3 months after**

- Feedback follow-up
- Action items follow-up

## Case Studies and Examples

Reviewing examples of prior faculty retreats in academic medicine can be particularly valuable and can demonstrate both areas of success and lessons learned. In 2022, Lee et al [36] published about their experience with the Abdominal Radiology Division at Harvard Medical School, Brigham and Women’s Hospital. Held in 2021, this retreat aimed to specifically address division stressors and challenges experienced during the COVID-19 pandemic. Twenty-eight faculty participated in a 2 and a half-hour virtual retreat via Zoom (Zoom Communications). A preretreat survey was used to establish discussion topics as well as determine current faculty satisfaction. A postretreat survey assessed retreat effectiveness and faculty satisfaction regarding stated objectives. With regard to limitations, it was suggested that the size of the larger group (N=28) may have caused hesitancy in discussion responders, and the authors also noted that the retreat was relatively short in duration. However, the authors ultimately concluded that the retreat was successful with regards to several outcomes, including promoting group camaraderie, provisioning ideas to improve the work environment (eg, adjusting shift times or coverage), and identifying specific faculty academic needs (eg, mentorship and sponsorship).

Birx et al [37] published their experience with a retreat held for nursing faculty at Radford University. Specifically designed within a positive psychology framework, the stated retreat goal was to build faculty relationships to create a cohesive team and to accomplish institutional goals. Held at a nature conservatory owned by the university and involving 29 nursing faculty, the daylong team-building event used challenge course activities, described as “an experiential adventure program…involving mental, physical, and emotional risk taking.” The authors used a mixed method evaluation, both pre- and post retreat, considering qualitative and quantitative data. They also incorporated an extended postevaluation, which was completed at the end of the semester to consider potential longer-term effects of the retreat. The authors concluded that the retreat offered an immediate positive effect on their targeted outcomes; however, these were not necessarily sustained throughout the semester, suggesting that a one-time intervention may not be sufficient to impart continued impact. This experience highlights the importance of building on the momentum achieved at a retreat and using it as a foundation for ongoing development.

While previously unpublished, the authors of this tutorial also recently planned and implemented a faculty retreat. Of note, a paucity of available free “how to” content to guide the authors was the impetus for this tutorial paper as well as a specific scholarly objective of the held retreat. The inaugural EmpowerED Faculty Retreat for women in the University of Alabama at Birmingham (UAB) Department of Emergency Medicine was held September 4‐6, 2024, at a resort and outdoor leisure destination. Designed to foster leadership development, professional growth, wellness, and team building among faculty, the retreat featured didactic sessions, small group discussions, and wellness activities in a reflective, off-campus setting. A postretreat survey, completed by 11 of the 12 participants, revealed high levels of satisfaction and strong support for continuing this initiative. All respondents agreed that the retreat met or exceeded their expectations, citing a well-balanced agenda that integrated learning, networking, and self-care. The workshops—Dare to Dialogue: Navigating Difficult Conversations and Microskills for Professional Success—were particularly well received. Nearly 3 quarters of attendees reported applying retreat content within 1 month, including strategies for communication, time management, and relationship-building. Participants praised the retreat location and logistics, noting that the peaceful setting supported creativity and deeper connection. However, several suggested that centralized lodging would have enhanced informal interaction, particularly in the mornings and between sessions. Feedback also highlighted a desire for more small group engagement, 1:1 mentoring opportunities, and dedicated time to discuss department-specific topics. Looking ahead, faculty expressed strong interest in future retreats, identifying topics, such as negotiating, attaining promotion, and mentorship as high priorities. The most favored strategies for sustaining the work of the retreat included annual offsite gatherings, quarterly check-ins, and peer mentoring. Overall, the EmpowerED retreat was viewed as a transformative and affirming experience, supporting both individual development and a stronger sense of community within the department.

## Future Directions and Emerging Trends

As models of faculty development, including retreat incorporation, continue to expand and evolve, several recent trends have been noted. These trends align with the current state of medicine, reflecting key developments, such as the emphasis on clinician well-being and integrating the growth of advanced technologies in both clinical practice and academic settings.

The COVID-19 pandemic was associated with a record-high physician burnout rate. While the American Medical Association (AMA) reports that these numbers have improved more recently, percentage rates remain astonishingly high, over 50% for some subspecialties [38]. There has been a noted emphasis on integrating wellness, mindfulness, resiliency training, and structured time for reflection into today’s faculty and trainee retreats [32,39].

Considering advanced technology, faculty development can both use and integrate content specific to emerging technology, particularly artificial intelligence (AI), data analytics, and virtual platforms, which offer new opportunities to enhance the planning, delivery, and impact of faculty retreats. AI-driven tools may be used to streamline various aspects of retreat implementation. For example, natural language processing tools can analyze preretreat survey responses to identify common themes and tailor agendas to faculty needs. AI scheduling assistants can coordinate availability across large faculty groups, reducing the administrative burden on organizers. During the retreat itself, AI-powered collaboration platforms (eg, Miro with AI clustering features or Microsoft Copilot) can help synthesize live input from participants, identify emerging priorities, and generate summaries in real time. In the realm of personalized learning, AI-enabled learning management systems can recommend postretreat faculty development resources aligned with individual career goals, teaching portfolios, or scholarly interests. These platforms may also track progress and provide nudges for continued engagement, thereby extending the retreat’s value over time.

Virtual and hybrid retreat models, initially born out of necessity, now offer sustainable and scalable solutions for faculty development—particularly in geographically dispersed departments. With the integration of AI-enhanced virtual facilitators, breakout room optimization, and sentiment analysis, retreat organizers can gain immediate feedback and adjust facilitation strategies accordingly. When appropriate, a hybrid approach to a retreat (combining in-person and virtual participation) might allow for greater flexibility, particularly when inviting outside speakers. Virtual or hybrid models may also have the added benefit of decreasing overall budget. When considering the incorporation of virtual components, it is important to ensure that the audiovisual technology is high-functioning, and a moderator (in person, if hybrid) should facilitate virtual speakers [40]. As these technologies continue to mature, their thoughtful application will be critical to ensuring that innovation complements, rather than replaces, the relational and reflective core of faculty retreats. Further, a careful consideration of outcome comparisons in virtual versus hybrid versus in-person retreats should be a consideration for future analysis.

## Conclusion

In conclusion, professional development for academic medicine faculty is a goal-directed, continuous effort pursued over one’s entire career span and is vital to improve and hone skills. Faculty retreats, an increasingly popular form of professional development, can lead to improved faculty performance, effectiveness, and wellness. Initial retreat planning should include a needs assessment from the targeted audience, the results of which should be used to define retreat objectives. Then, SMART goals should be set to guide the retreat’s agenda and outcomes. In order to meet retreat objectives, other important considerations include resource allocation (including personnel coordination and oversight) and participant selection, as well as location and setting choice. Following the event, solicited postretreat feedback can translate positive retreat outcomes into ongoing faculty improvement and development. Faculty retreats can represent a diverse, expansive, and valuable development tool for academic medicine. Careful planning consideration and subsequent implementation can help ensure their success.

## Supplementary material

10.2196/71622 Multimedia Appendix 1 Example postretreat assessment.

## Abbreviations

AI: artificial intelligence
AMA: American Medical Association
SMART: Specific, Measurable, Achievable, Relevant, and Time-bound
UAB: University of Alabama at Birmingham
