# Supplementary material for: Faculty Retreats in Academic Medicine: Tutorial
Source: JMIR Med Educ. 2025 Nov 3;11:e71622. doi: 10.2196/71622 (PMC12582542; doi:10.2196/71622)
Supplement: Multimedia Appendix 1 [file mededu-v11-e71622-s001.docx]

*Multimedia Appendix 1.* Example Post-Retreat Assessment

**Emergency Medicine Faculty Retreat**
*Thank you for participating in our recent faculty retreat. Your feedback is essential to help us improve future events and ensure they continue to meet your needs.*

**Section 1: Retreat Content & Format**

**1. Please rate the following aspects of the retreat:**
(Scale: 1 = Poor, 2 = Fair, 3 = Good, 4 = Very Good, 5 = Excellent)

| **Component** | **Rating** |
| --- | --- |
| Overall retreat experience | ☐ 1 ☐ 2 ☐ 3 ☐ 4 ☐ 5 |
| Relevance of session topics | ☐ 1 ☐ 2 ☐ 3 ☐ 4 ☐ 5 |
| Balance between structured sessions and open time | ☐ 1 ☐ 2 ☐ 3 ☐ 4 ☐ 5 |
| Usefulness of workshops/breakouts | ☐ 1 ☐ 2 ☐ 3 ☐ 4 ☐ 5 |
| Effectiveness of facilitators/speakers | ☐ 1 ☐ 2 ☐ 3 ☐ 4 ☐ 5 |
| Opportunities for interaction and engagement | ☐ 1 ☐ 2 ☐ 3 ☐ 4 ☐ 5 |

**Section 2: Outcomes & Impact**

**2. To what extent do you agree with the following statements?**
(Scale: Strongly Disagree – Disagree – Neutral – Agree – Strongly Agree)

- I gained new insights or knowledge applicable to my role.
- I feel more connected to my colleagues after the retreat.
- The retreat enhanced my understanding of departmental priorities.
- I left the retreat with concrete actions I plan to take.
- I would recommend participating in future retreats.

**Section 3: Open-Ended Feedback**

**3. What was the most valuable part of the retreat for you?**
*____________________________________________________*

**4. What could be improved for future retreats?**
*____________________________________________________*

**5. Were there any topics or issues you felt were missing or underexplored?**
*____________________________________________________*

**6. Do you have ideas for future retreat themes, sessions, or speakers?**
*____________________________________________________*

**Section 4: Follow-Up and Next Steps (Optional)**

**7. Would you be interested in participating in follow-up working groups related to retreat themes (e.g., mentoring, wellness, research)?**
☐ Yes ☐ No ☐ Maybe

**8. Any additional comments or reflections?**
*____________________________________________________*
